# Safety and Efficacy of Exclusive Enteral Nutrition for Percutaneously Undrainable Abdominal Abscesses in Crohn's Disease

**DOI:** 10.1155/2017/6360319

**Published:** 2017-08-30

**Authors:** Yibin Zhu, Liang Xu, Wei Liu, Weilin Qi, Qian Cao, Wei Zhou

**Affiliations:** ^1^Department of General Surgery, Sir Run Run Shaw Hospital, School of Medicine Zhejiang University, Hangzhou, China; ^2^Inflammatory Bowel Disease Center, Sir Run Run Shaw Hospital, School of Medicine Zhejiang University, Hangzhou, China; ^3^Department of Gastroenterology, Sir Run Run Shaw Hospital, School of Medicine, Zhejiang University, Hangzhou, China

## Abstract

**Background:**

The percutaneously undrainable abdominal abscesses in Crohn's disease (CD) are not uncommon. The treatment protocol is still under debate. This study was conducted to assess the safety and efficacy of exclusive enteral nutrition (EEN) for percutaneously undrainable abscesses in CD.

**Methods:**

A consecutive cohort of 83 CD patients with percutaneously undrainable abdominal abscesses between January 2011 and June 2015 was retrospectively analyzed. They were divided into the EEN group and the non-EEN group.

**Results:**

The cumulative surgical rate was significantly lower in the EEN group than in the non-EEN group (*P* = 0.001). Fifteen percent patients treated with EEN avoided surgery. EEN (*P* = 0.002) was associated with a decreased need for surgery. Previous abdominal surgery (*P* = 0.009) and abscess diameter > 3 cm (*P* = 0.022) were associated with an increased need for operation. EEN increased the albumin level, while decreased ESR and CRP significantly for patients requiring surgery. The risk of postoperative intra-abdominal septic complications (*P* = 0.036) was significantly lower in the EEN group compared with the non-EEN group.

**Conclusions:**

EEN is feasible in CD patients presenting with percutaneously undrainable abdominal abscesses. It is associated with a reduction in surgical rate, optimized preoperative condition, and improved postoperative outcomes in these specific groups of patients.

## 1. Introduction

Crohn's disease (CD) is a chronic inflammatory disorder of the intestine including nonstricturing/nonpenetrating, stricturing, and penetrating disease phenotypes [1]. Penetrating disease may be complicated by abscess formation. This complication accounts for the high rate of surgery in patients with CD. In the literature, occurrence rates for abdominal abscesses vary from 10 to 30% [2]. The common treatments include antibiotics, percutaneous or surgical drainage of fluid collections, bowel rest, and, eventually, resection of the diseased bowel. Nowadays, percutaneous drainage (PD) has been served as a bridging strategy before definitive surgery to reduce postoperative complications [3]. Patients with concomitant stenosis, enterocutaneous fistula, or refractory active disease are likely to require surgery.

Though PD has been increasingly used for well-defined and unilocular abscesses, occasionally, abscesses are not technically drainable for PD due to the difficult location or multilocular form [4], such as the patients with mesenteric and interloop abscesses, which were difficult to treat with PD. Under these circumstances, only antibiotics may be prescribed for patients, while steroids, immunosuppressants, and antitumor necrosis factor (anti-TNF) agents are avoided because of the potential for peritoneal and systemic infection [5]. For patients who do not respond to antibiotics, definite surgery or a defunctioning ostomy to cool down the abscesses is usually needed [6]. The patients then take the risk of more postoperative complications or repeated operations.

Exclusive enteral nutrition (EEN) is considered to be of great importance in patients with CD, which provided the liquid nutrition formula to meet the daily nutritional requirements. In children with CD, the EEN is the first line therapy to treat active CD instead of corticosteroid [7, 8]. EEN has shown to be an effective and safe treatment option to induce remission of CD in adults [9]. A 4-week EEN treatment has the effect of improving health-related quality of life in adults with active CD [9]. However, use of EEN in patients with abscesses remains controversial because of the bowel rest principle for penetrating CD treatment. Only a few studies have formally evaluated the clinical outcomes of EEN in penetrating disease [10–12]. As far as we know, there were no published studies in the current literature which determined the effect of EEN on outcomes in CD patients with percutaneously undrainable abscesses. Therefore, the aim of this retrospective study was to specifically evaluate the following: (1) the safety of EEN for abdominal abscesses in CD, (2) the surgical rate of these patients on EEN, and (3) the effect of EEN on preoperative optimization and postoperative outcomes.

## 2. Materials and Methods

### 2.1. Patients and Definitions

All the charts of consecutive CD patients from January 2011 to June 2015 in Sir Run Run Shaw Hospital were reviewed. An abscess was defined as an extraluminal ring enhancing fluid collection 1 cm or greater in diameter, identified on computed tomography (CT) or magnetic resonance imaging (MRI) [13]. Abscesses developed within one month after abdominal surgery were considered as postoperative complications and excluded. Accessibility of PD was discussed with surgeons and radiologists. CT or MRI was used to assess whether the abscess was subsided or not after treatment.

### 2.2. Patient Management

Treatments for the percutaneously undrainable abscesses were generally started with nutrition management and concomitant antibiotics. In the routine clinical practice at our inflammatory bowel disease (IBD) center, the patients presenting with percutaneously undrainable abdominal abscesses are recommended with EEN treatment. However, some patients failed to receive the EEN therapy because they can not tolerate the EEN or comply with the EEN therapy. Thus, in our study, the patients with well tolerance with the EEN or good compliance with the EEN therapy were assigned to the EEN group, while the patients with poor compliance or poor tolerance with the EEN therapy were assigned to the non-EEN group.

The EEN administered was a product of the Nutricia company (Wuxi, China) called Peptison liquid, which is composed of maltodextrin, whey protein hydrolysate, citric acid, vegetable oil, medium-chain triglyceride, vitamins, minerals, and trace elements. The content of the three major nutrients included 17.6 grams carbohydrate, 1.7 grams lipid, and 4 grams of protein per 100 mL liquid. The calorie density was 1 kcal/mL with an osmolarity of 440 mosm/L. The elemental formula was taken continuously through a nasogastric tube by using an infusion pump. The dosage of EEN was gradually increased to the full dose in order to reduce side effects such as diarrhea and abdominal distension. The patients were recommended to use of EEN until abscess resolution or surgery. The daily calorie was 25–30 kcal/kg body weight. During the period of EEN, any other food or drink was forbidden except water. Antibiotics were discontinued when the patients' temperature and white blood cell count normalized. Surgery was indicated when the patients had no clinical improvement, persistent or recurrent abscesses, fistulas, or bowel obstruction.

### 2.3. Data Collection

The electronic medical records were accessed to retrieve the following information on eligible subjects: demographic data, date of CD diagnosis, date of abscess diagnosis, location and size of abscess, multiplicity of abscess, Montreal classification [1], previous CD-related abdominal surgery, laboratory and radiology tests, concurrent medical therapy, compliance of EEN therapy, abscess resolution rates, time to surgery, surgical procedure, stoma creation, postoperative complications, length of hospital stay after surgery, and length of follow-up. Postoperative intra-abdominal septic complications (IASCs) were defined as any anastomotic leak, intra-abdominal abscess, or enterocutaneous fistula that occurred within the first 30 days after surgery [14]. The primary outcome was the subsequent surgery. Secondary endpoints were postoperative IASCs and duration of hospitalization.

This study was approved by the institutional review board at Sir Run Run Shaw Hospital and was conducted in accordance with the Helsinki Declaration.

### 2.4. Statistical Analysis

Statistical analysis was performed with SPSS for Windows, version 19.0 (SPSS; Chicago, IL). Quantitative data were expressed as mean ± SD or medians with ranges. Comparisons between groups were conducted using Student's *t*-test, Fisher's, or *χ*^2^ test. The cumulative surgical rate was estimated by the Kaplan-Meier approach and compared using the log-rank test. The predictors for surgery were evaluated by multivariate analysis using a Cox proportional hazards model. A *P* < 0.05 was considered statistically significant.

## 3. Results

### 3.1. Patient Characteristics

Clinical characteristics of patients enrolled in the study are indicated in Table 1. According to the enroll criterion, we identified 83 patients with CD presenting with abscesses, which were considered technically unsuitable to PD because of the multilocular form or unfavorable abscess location with an extremely difficult or impossible access pathway to the abscess cavity. The mean age was 34.33 ± 11.06 years, ranging from 17 to 60 years. 47 of 83 (56.6%) were males. The duration of CD ranged from 36 days to 30 years (median, 4.73 years).

According to Montreal classification, disease phenotypes were ileocolonic in 52 (62.65%), colonic in 3 (3.61%), and jejunoileal in 28 (33.73%) patients. Previous CD-related abdominal surgery was reported in 19 (22.89%) patients. According to the group definition, 54 patients were in the EEN group, and 29 patients were in the non-EEN group. The mean duration of EEN treatment was 5.9 weeks, ranging from 2 to 8 weeks. There were no severe adverse events that happened in both group. Patients' characteristics were mostly comparable in the EEN group and the non-EEN group as shown in Table 1. However, C-reactive protein (CRP) levels (*P* = 0.029) and white blood cell (WBC) levels (*P* = 012) were significantly higher in the EEN group compared with the non-EEN group.

### 3.2. Cumulative Surgical Rate

**Figure 1:**
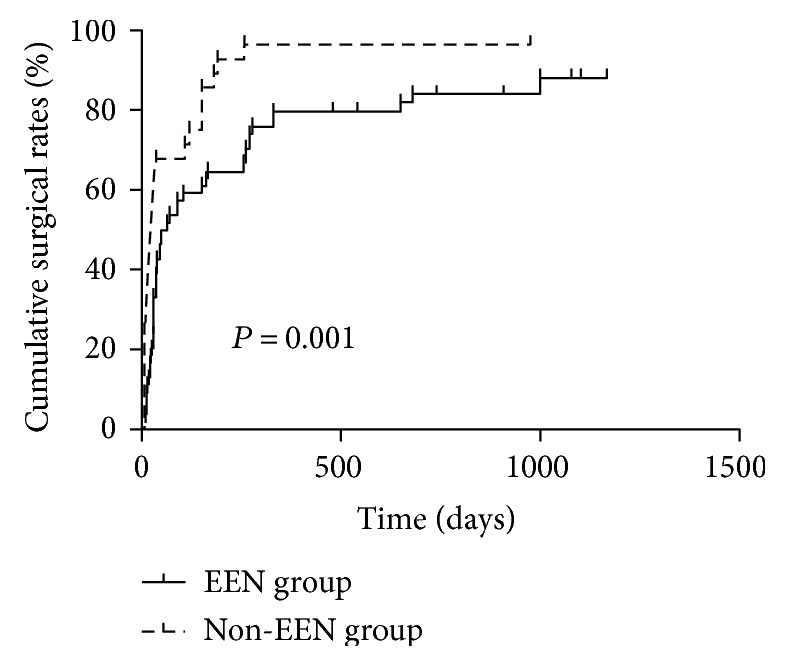
The cumulative surgical rate was significantly lower in the EEN group than in the non-EEN group (*P* = 0.001).

The cutoff date of the follow-up for all participants was on January 31, 2017. The median follow-up period for the entire study group was 19.8 months, ranging from 3.0 to 65.3 months. There were no serious adverse events reported in all patients with EEN treatment. During the follow-up period, the estimated cumulative rates of surgery for CD patients with undrainable abscess were 43% at 1 month, 61% at 3 months, 85% at 1 year, and 90% at 2 years. The cumulative surgical rate was significantly lower in the EEN group than in the non-EEN group (*P* = 0.001, Figure 1). By the end of follow-up, 46 patients in the EEN group (78.8%) and 28 patients in the non-EEN group (92.3%) required surgery. Among which, 41 patients in the EEN group and 19 patient in the non-EEN group had abscess resolution (*P* = 0.023). The median length of time between onset of abscess and surgery was 39 days (range, 7~1000 days). 8 patients in the EEN group and 1 patient in the non-EEN group avoided surgery by the end of follow-up.

### 3.3. The Clinical Factors Associated with Surgery

The effects of categorical variables on the overall time to surgery were analyzed with the Kaplan-Meier method. The results of the log-rank tests for each categorical variable are presented in Table 2. Univariate analysis identified that EEN was associated with a decreased need for operation and 2 clinical variables associated with an increased need for operation: previous abdominal surgery and abscess diameter > 3 cm. Age, gender, perianal disease, fistula, disease location and administration of steroids and immunosuppressants at the time of abscess diagnosis were not predictors for surgery.

Table 3 shows results of the final multivariable Cox proportional hazards regression analysis for surgery. All variables were fit in the same multivariable model. In this model, EEN (HR, 0.461; 95% CI, 0.282–0.753; *P* = 0.002) was associated with a decreased need for operation. Previous abdominal surgery (HR, 2.069; 95% CI, 1.203–3.556; *P* = 0.009) and abscess diameter > 3 cm (HR, 1.831; 95% CI, 1.092–3.071; *P* = 0.022) were significantly associated with an increased need for surgery.

### 3.4. Changes of the Serum Parameters and Outcomes in Patients Undergoing Surgery

**Figure 2:**
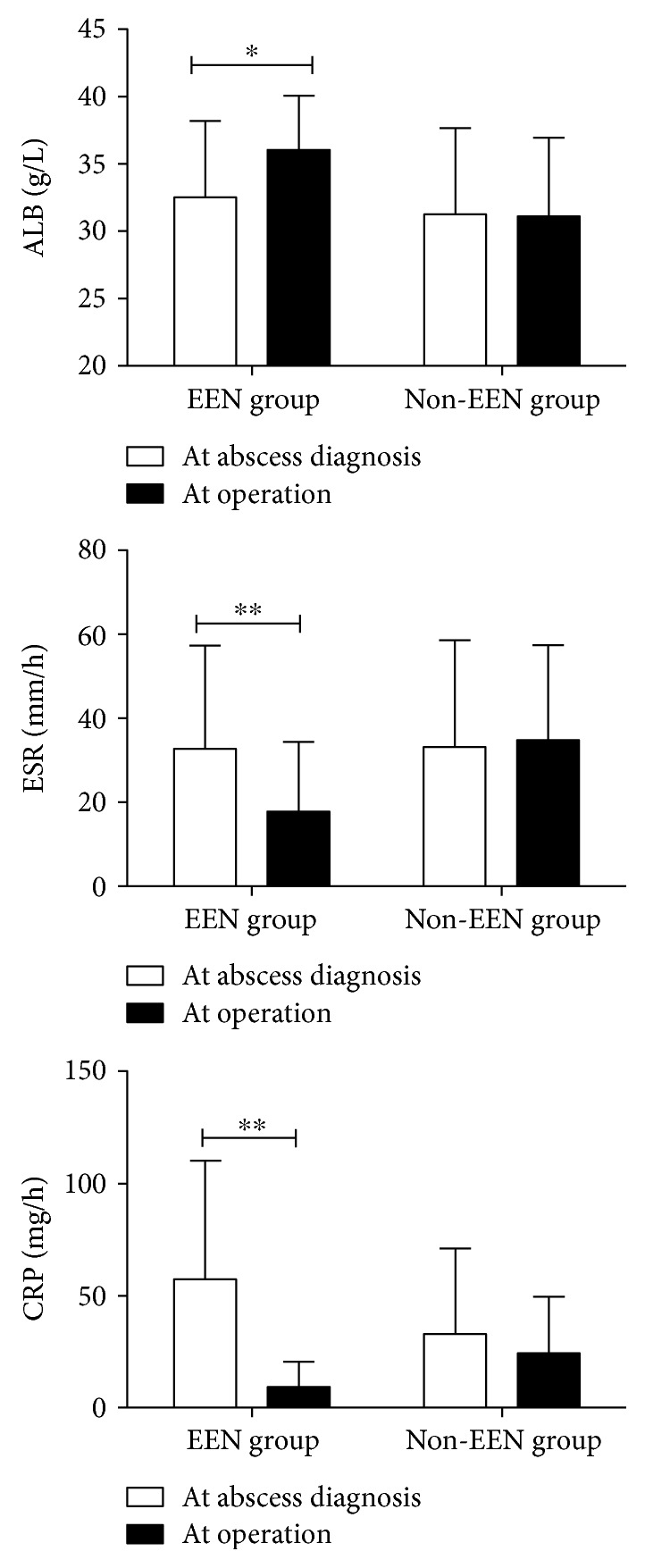
Comparison of the levels of albumin, erythrocyte sedimentation rate (ESR), and C-reactive protein (CRP) at abscess diagnosis and at operation in both groups of patients who underwent surgery later (^∗^*P* < 0.05, ^∗∗^*P* < 0.001).

Then, we compared the levels of albumin, erythrocyte sedimentation rate (ESR), and C-reactive protein (CRP) at abscess diagnosis and at operation in both groups of patients who underwent surgery later. In the EEN group, the albumin level increased while ESR and CRP decreased remarkably after receiving preoperative EEN. In the non-EEN group, there were no significant differences of these parameters (Figure 2, ^∗^*P* < 0.05, ^∗∗^*P* < 0.001). We then investigated the differences of operative time, blood loss, stoma creation, postoperative IASCs, and postoperative hospital stay between the EEN and the non-EEN groups. The result shows that the risk of postoperative IASCs (*P* = 0.036) and duration of postoperative hospitalization (*P* = 0.032) were significantly decreased in the EEN group compared with the non-EEN group for patients undergoing surgery (Table 4).

## 4. Discussion

In the present study, we found that EEN could be safely used in CD patients with percutaneously undrainable abdominal abscesses. It has both the anti-inflammatory and nutritional effects, which may lead to abscess resolution and preoperative optimization and even avoidance of surgery.

The management strategy for spontaneous CD-related abdominal abscesses has traditionally been PD followed by elective surgery treating the underlying diseased intestine. Inaccessibility of abscess for image-guided PD made this intervention inapplicable in some patients. In the setting, antibiotics alone seems only effective for small abscesses without associated fistula [15]. Most of the patients who failed the PD require surgery after a short period of time [16]. Unfortunately, the overall complication rate was significantly higher in patients undergoing initial or urgent surgery compared with those undergoing initial PD [17].

Enteral nutrition, a cheaper and safer treatment strategy, could be offered as an alternative option to treat CD [18]. EEN is able to reduce CD activity and maintain remission in both adults and children [7–9, 19]. Numerous studies have found that preoperative EEN effectively relieves inflammatory bowel stricture and reduces the postoperative septic complications of fistulizing CD [11, 20]. Li et al. [11] have found that the serum albumin level increases while CRR decreases remarkably in patients receiving preoperative EEN, which is in accordance with our findings. In a recent study, Heerasing et al. [10] found that EEN have the effect of decreasing the need for surgery in patients presenting with stricturing or penetrating complications of CD disease and also have the value of reducing systemic inflammation and the incidence of postoperative IASCs. The results were also consistent with our finding. However, previous studies on the safety and efficacy of EEN in CD patients with abdominal abscesses are limited. We found here that EEN could optimize the patients' condition by improving the nutritional parameter, suppressing the inflammatory parameters. In addition, EEN postponed the definite surgery, improved the postoperative outcome and even avoided surgery.

Abscesses are usually associated with fistula or perforating complications, which leads to transmural translocation of bacteria from the diseased bowel to the contiguous tissue. Classically, for patients with abscess, oral feeding should be stopped to allow bowel rest, and logically conservative treatment with parenteral nutrition has been proposed as supportive therapy until the acute inflammation episode begins to resolve. In this situation, concern is raised about the aggravation of abdominal infection by enteral feeding. However, we demonstrate that EEN in patients with Crohn's abscess is safe. Most of the patients in the EEN group tolerated EEN, with only minor complications of diarrhea and abdominal floating in several cases. Fistulas were more common in the EEN group than in the non-EEN group, though the difference was not significant. In a previous study, fistula presence was associated with poor PD outcomes [21]. However, our results suggest that EEN is safe and tolerable in CD patients with concomitant abscess and fistula. It has the effects of both abscess resolution and concrescence of the fistula.

The precise mechanisms underlying the effect of enteral nutrition to cool down abscess are poorly understood. It might be multifactorial involving anti-inflammatory effects, mucosal healing stimulation, and intestinal microbiota modification [22, 23]. Apparently, a good nutritional status would theoretically lead to enhanced mucosal healing and systemic immune response [24]. Enteral nutrition has been shown to decrease intestinal permeability, which is associated with pathogenesis of CD [25]. Reduction in the workload of digestion and absorption by enteral nutrition may also play a role. Enteral nutrition might also change the intestinal flora participating in the development of inflammation in CD, thus lowering the risks of bacterial translocation and subsequent infection [26].

Final Cox proportional hazards model in our study showed that abscess size >3 cm and previous bowel resections increased the risk for surgery. This is not surprising. Large abscesses are always associated with more severe bowel diseases, leading to bowel obstruction and refractory abscesses. Riss et al. also found that previous resections for CD were significant risk factors for reoperation primarily because of the more aggressive disease pattern in this group of patients [27].

There are also some limitations in our study. First, this was a retrospective study and the number of patients in this study was relatively small. Despite this, our results represent the only report on the use of EEN in CD complicated by percutaneously undrainable abdominal abscess. Obviously, further and larger prospective studies are required to verify the results. Secondly, there have been some patients that received the concomitant biological or immunosuppressant therapy after abscess resolution, which may have an impact on the patients' long-term outcomes, especially the rate of surgery. Despite this, the 1-year cumulative surgical rate was also significantly lower in the EEN group than in the non-EEN group. Finally, there have been a small number of patients that have withdrawn the EEN treatment before the surgery although all of them had abscess resolution, which may affect the patients' perioperative status and the surgical outcomes. Thus, a larger prospective study is required to define the optimal time interval between EEN and surgery in patients with abdominal abscesses.

In conclusion, the results of our study confirm that EEN is the treatment of choice in patients with percutaneously undrainable abdominal abscesses in CD. It is effective to resolve abscess, improve nutritional status, and cool down the inflammation. Such a protocol is also associated with a reduced surgical rate and optimized preoperative condition as well as improved postoperative outcomes.

## Figures and Tables

**Table 1:** Demographic and clinical characteristics of CD patients with percutaneously undrainable abscesses by the treatment group.

Characteristics	EEN group (*n* = 54)	Non-EEN group (*n* = 29)	*P*
Gender, *n* (male/female)	32/22	15/14	0.509
Age at onset of abscess (y), median (range)	32.8 (17–51)	37.1 (17–60)	0.094
Disease duration (y), median (range)	4.3 (0.16–30)	5.5 (0.1–20)	0.274
Previous abdominal surgery, *n* (%)	11 (20.4)	8 (27.6)	0.456
Montreal classification of age, *n* (%)			0.526
A1 (<16 years)	0 (0)	0 (0)	
A2 (17–40 years)	39 (72.2)	19 (65.5)	
A3 (>40 years)	15 (27.8)	10 (34.5)	
Montreal classification of disease location, *n* (%)			1.000
L1 (ileal)	18 (33.3)	10 (34.5)	
L2 (colonic)	2 (3.7)	1 (3.4)	
L3 (ileocolonic)	34 (63.0)	18 (62.1)	
Abscess location, *n* (%)			0.574
Right lower quadrant	24 (44.4)	13 (44.8)	
Pelvis	18 (33.3)	7 (24.1)	
Others	12 (22.2)	9 (31.0)	
Multiple abscesses, *n* (%)	8 (14.8)	7 (24.1)	0.293
Abscess diameter > 3 cm, *n* (%)	11 (11.1)	11 (24.1)	0.084
Fistula, *n* (%)	31 (57.4)	11 (37.9)	0.091
Perianal lesion, *n* (%)	7 (13.0)	7 (24.1)	0.227
Steroids at diagnosis, *n* (%)	18 (33.3)	14 (48.3)	0.182
AZA/6-MP at diagnosis, *n* (%)	20 (37.0)	14 (48.3)	0.321
Infliximab at diagnosis, *n* (%)	7 (13.0)	3 (10.3)	1.000
Laboratory indices			
White blood cell (×10^9^/L), mean, (range)	8.8 (3.6–15)	7.1 (2.7–11.6)	0.012
Hemoglobin (g/L), mean (range)	11.8 (6.1–15.8)	10.9 (6.6–14.1)	0.366
ESR (mm/h), mean (range)	36.7 (2–92)	33.3 (2–96)	0.574
C-reactive protein (mg/L), mean (range)	56.4 (3.9–202.4)	32.6 (0.4–159)	0.029
Albumin (g/L), mean (range)	32.5 (19.5–41.1)	30.8 (17–40.3)	0.216

ESR: erythrocyte sedimentation rate; EEN: exclusive enteral nutrition; AZA: azathioprine; 6-MP: mercaptopurine.

**Table 2:** Univariate analysis of potential predictors for surgery in CD patients with percutaneously undrainable abscesses.

Variable	1-year cumulative surgical rate (%)	*P* (log-rank)
Gender		0.972
Male	87.2	
Female	83.3	

Age at diagnosis		0.895
A1 (16 years)	0	
A2 (17–40 years)	86.2	
A3 (40 years)	84.0	

Disease location		0.208
L1 (ileal)	85.7	
L2 (colonic)	100.0	
L3 (ileocolonic)	84.6	

Steroids use at diagnosis		0.703
No	86.3	
Yes	84.4	

AZA/6-MP use at diagnosis		0.550
No	89.8	
Yes	79.4	

Infliximab use at diagnosis		0.360
No	87.7	
Yes	70.0	

Perianal lesion		0.922
No	85.5	
Yes	85.7	

Previous abdominal surgery		0.007
No	81.2	
Yes	100.0	

Abscess diameter > 3 cm		0.006
No	80.3	
Yes	100	

Fistula		0.928
No	85.4	
Yes	85.7	

Multiple abscesses		0.060
No	82.4	
Yes	100	

Abscess location		0.253
Right lower quadrant	83.8	
Pelvis	88.0	
Others	85.7	

Exclusive enteral nutrition		0.001
No	96.6	
Yes	79.6	

AZA: azathioprine; 6-MP: mercaptopurine.

**Table 3:** Multivariable Cox regression analysis of clinical factors associated with surgery in CD patients with percutaneously undrainable abscesses.

Variable	Model coefficient	Hazard ratio (HR)	95% CI	*P* value
Abscess diameter > 3 cm	0.605	1.831	1.092–3.071	0.022
Previous abdominal surgery	0.727	2.069	1.203–3.556	0.009
Exclusive enteral nutrition	−0.775	0.461	0.282–0.753	0.002

**Table 4:** Characteristics and postoperative short-term outcomes of patients requiring surgery.

Characteristics and outcomes	EEN group (*n* = 46)	Non-EEN group (*n* = 28)	*P*
Gender, *n*			0.537
Male	28	15	
Female	18	13	

Montreal classification of age at abscess diagnosis, *n*			0.723
A1 (<16 years)	0	0	
A2 (17–40 years)	33	19	
A3 (>40 years)	13	9	

Montreal classification of disease location, *n*			0.912
L1 (ileal)	15	10	
L2 (colonic)	2	1	
L3 (ileocolonic)	29	17	

Previous surgical history, *n*	11	8	0.656

Multiple abscesses, *n*	8	7	0.430

Abscess location, *n*			0.636
Right lower quadrant	20	13	
Pelvis	16	7	
Others	10	8	

Perianal lesion, *n*	6	7	0.219

Abscess diameter > 3 cm, *n*	11	11	0.161

Fistula	29	10	0.022

Steroids at diagnosis, *n*	14	14	0.092

AZA/6-MP at diagnosis, *n*	15	14	0.137

Infliximab at diagnosis, *n*	6	3	1.000

Surgical approach			0.235
Open	15	13	
Laparoscopy	31	15	

Pre-op laboratory indices			
White blood cell (×10^9^/L), mean (range)	6.45 (2.4–24.4)	6.28 (3.8–14.9)	0.836
Hemoglobin (g/L), mean (range)	11.93 (7.4–14.5)	11.18 (7.1–12.7)	0.100
ESR (mm/h), mean (range)	17.82 (0–74)	34.86 (5–72)	<0.001
C-reactive protein (mg/L), mean (range)	8.704 (0–59.6)	23.80 (1.2–112.1)	0.001
Albumin (g/L), mean (range)	36.05 (28.4–42.5)	31.11 (23.1–41.3)	<0.001

Abscess resolution, *n*	41	19	0.023

Post-op IASCs, *n*	3	7	0.036

Stoma creation, *n*	20	13	0.804

Postop hospital stay (days), mean (±SD)	9.65 ± 6.75	13.64 ± 8.84	0.032

Operative time (min), mean (±SD)	203.63 ± 69.04	198.57 ± 52.38	0.740

Intraoperative bleeding (ml), mean (±SD)	87.94 ± 67.94	86.07 ± 57.18	0.904

IASCs: intra-abdominal septic complications; EEN: exclusive enteral nutrition; ESR: erythrocyte sedimentation rate; SD: standard deviation; AZA: azathioprine; 6-MP: mercaptopurine.
